# Assessing crystallisation behaviour in molecular crystals through particle rugosities

**DOI:** 10.1038/s42004-026-02104-5

**Published:** 2026-06-29

**Authors:** Marta Brocca, Dominic Evans, Helen Blade, Sten O. Nilsson Lill, Aurora J. Cruz-Cabeza

**Affiliations:** 1 Department of Chemistry, University of Durham, Durham, UK; 2 Global Product Development, Pharmaceutical Technology & Development, Operations, AstraZeneca Macclesfield, Cheshire, UK; 3 Predictive Science, Digital & Automation, Pharmaceutical Sciences, R&D, AstraZeneca Gothenburg, Mölndal, Sweden

**Keywords:** Computational chemistry, Drug discovery and development, Surface chemistry, Physical chemistry, Cheminformatics

## Abstract

Surface properties of molecular crystals play a central role in determining their nucleation, growth, and overall crystallisation behaviour. In particular, surface rugosity has been suggested as a meaningful descriptor of how readily a crystal may nucleate and grow, but the existing rugosity metrics have been limited by incomplete descriptions of surface topology. In this study, we present a new workflow for the computation of surface descriptors across crystal facets. Here the surface rugosity description for each facet is based on the Surface Area Ratio (SAR) definition proposed in the Cambridge Structural Database (CSD) tools. Given a crystal structure, our algorithm computes an average overall particle rugosity with three offset selection criteria that account for different crystallisation conditions. We then calculate particle rugosities and analyse data for a range of systems, including: polymorphic families, datasets from the CSD, and crystal structure prediction (CSP) landscapes. Our analysis shows that, in most cases, polymorphs with lower rugosities tend to nucleate and grow more readily, suggesting that this metric can distinguish experimentally accessible forms from more elusive ones. These findings demonstrate that particle rugosities can serve as a complementary tool for predicting and classifying the experimental feasibility of polymorphs generated computationally.

## Abstract (web summary)

Surface properties of molecular crystals play a central role in determining their nucleation, growth, and overall crystallisation behaviour, yet current rugosity metrics inadequately describe surface topology. Here, the authors introduce a workflow using surface area ratio to compute surface descriptors, revealing that lower rugosities correlate with easier nucleation and growth, proposing that particle rugosities can assist with classifying the experimental feasibility of predicted polymorphs.

## Introduction

Surfaces of molecular crystals exhibit remarkable structural diversity and chemical complexity because intermolecular interactions impose strong directionality on crystal packing. This directional packing defines distinct crystallographic orientations, each associated with specific molecular arrangements and interaction motifs. As a result, different crystal faces expose different surface terminations, revealing distinct functional groups, packing geometries, and local environments. Consequently, each symmetry-independent (hkl) surface presents a unique chemical composition and topography, leading to markedly different surface structures and interfacial properties.

Surface diversity is further amplified by the position along a given crystallographic direction at which the crystal is cleaved, referred to as the surface offset. The offset is defined relative to a reference (hkl) plane in the bulk structure, and its periodicity is the interplanar distance ${d}_{({hkl})}$ so that translations by integer multiples of ${d}_{({hkl})}$ produce crystallographically equivalent terminations. This dependence on both face and offset results in significant variation in surface chemistry and structure, influencing properties such as surface energy, interfacial tension, and reactivity. The distribution of these surfaces in crystals obtained during crystallization is governed by the underlying intrinsic interactions within the crystal, but also by external conditions such as solvent type and supersaturation. Resulting interfaces not only determine particle morphology but also impact nucleation and growth behaviour, ultimately affecting the physical properties of crystalline powders of critical importance in pharmaceutical development (such as dissolution rate, flowability, wettability, and stickiness^1–6^).

Understanding and controlling surface structure, properties, and crystal morphology is therefore essential for designing pharmaceutical products with desired performance. However, describing the full range of surface structures for a given crystal is challenging. Surfaces differ not only in chemistry but also in molecular-scale topography, exhibiting features such as peaks, troughs, ridges, and grooves defined by the arrangement and orientation of surface-exposed molecules. This surface roughness can be quantified using topological surface rugosity descriptors, defined here as the degree of molecular-scale corrugation relative to an ideal flat surface.

The first computational approach to compute surface rugosities, introduced by Bryant et al.^7^, quantified them for individual (hkl) planes using a one-dimensional descriptor ${R}_{{\mbox{depth}}}^{\left({{\rm{hkl}}}\right)}$. This 1D distance descriptor measures the maximum atomic intrusion (in Å) between consecutive layers of a given (hkl), providing a measure of roughness and surface interlocking. This computation of surface rugosity was a useful means to explore crystallisation tendencies^8–10^ and mechanical properties of crystalline particles^11,12^.

Building on this descriptor, Montis et al.^13^ developed an average particle rugosity (${\bar{R}}_{{{\rm{depth}}}}^{{{\rm{BFDH}}}}$) metric for crystals. This descriptor normalises ${R}_{{\mbox{depth}}}^{\left({{\rm{hkl}}}\right)}$ by ${d}_{({hkl})}$ (to enable comparison across systems of different sizes) and returns an average normalised rugosity value for the overall crystal particle assuming BFDH-generated morphologies^14–16^ as a good predictor of crystal shape. The authors found a correlation between higher average particle rugosities with elusive crystallisation and polymorphism. While effective, the average particle rugosity descriptor computed with the normalised 1D ${R}_{{\mbox{depth}}}^{\left({{\rm{hkl}}}\right)}$ rugosity remains too simplistic to truly describe overall surface topographies and lacks any considerations of solvent effects. Nevertheless, these simple descriptors have demonstrated that overall surface rugosity of a crystal particle provides significant insights into crystallization behaviour^17,18^.

Conceptually, surface roughness influences how molecules interact with and attach to crystal surfaces, affecting both thermodynamics and kinetics of crystallisation. During nucleation, smooth surfaces typically offer more uniform interaction sites, reducing energetic penalties and lowering surface free energy^9,19^. During crystal growth, rough surfaces can hinder growth at low supersaturation due to solvent blocking growth sites^8^, but promote rapid growth under diffusion-controlled conditions at high supersaturation^10^. These observations highlight rugosity as a predictive descriptor of crystallization outcomes across diverse systems.

Recent development of more complex descriptors of (hkl) surface rugosities have now been implemented in the Cambridge Structural Database (CSD)^20^ particle tool, CSD-Particle^21^. Amongst them, the 3D surface topology descriptor Surface Area Ratio (SAR) describes the ratio between the real surface area (SA) of an (hkl) surface over its SA projection. Building on these developments, we present a Python-based algorithm to compute average particle rugosity (${\bar{R}}_{{\mbox{SAR}}}$) using this SAR descriptor as defined by the CSD, also incorporating multiple morphology generation methods and possible solvent effects. We apply this approach to six varied datasets, including polymorphic families, CSD datasets, and CSP landscapes, demonstrating that lower ${\bar{R}}_{{\mbox{SAR}}}$ values correlate with more readily crystallizable forms. Our findings establish particle rugosity as a powerful complement to existing polymorph prediction and screening strategies and provide a quantitative link between molecular-level surface structure and macroscopic crystallization outcomes.

## Methods

### Definitions

**Fig. 1:**
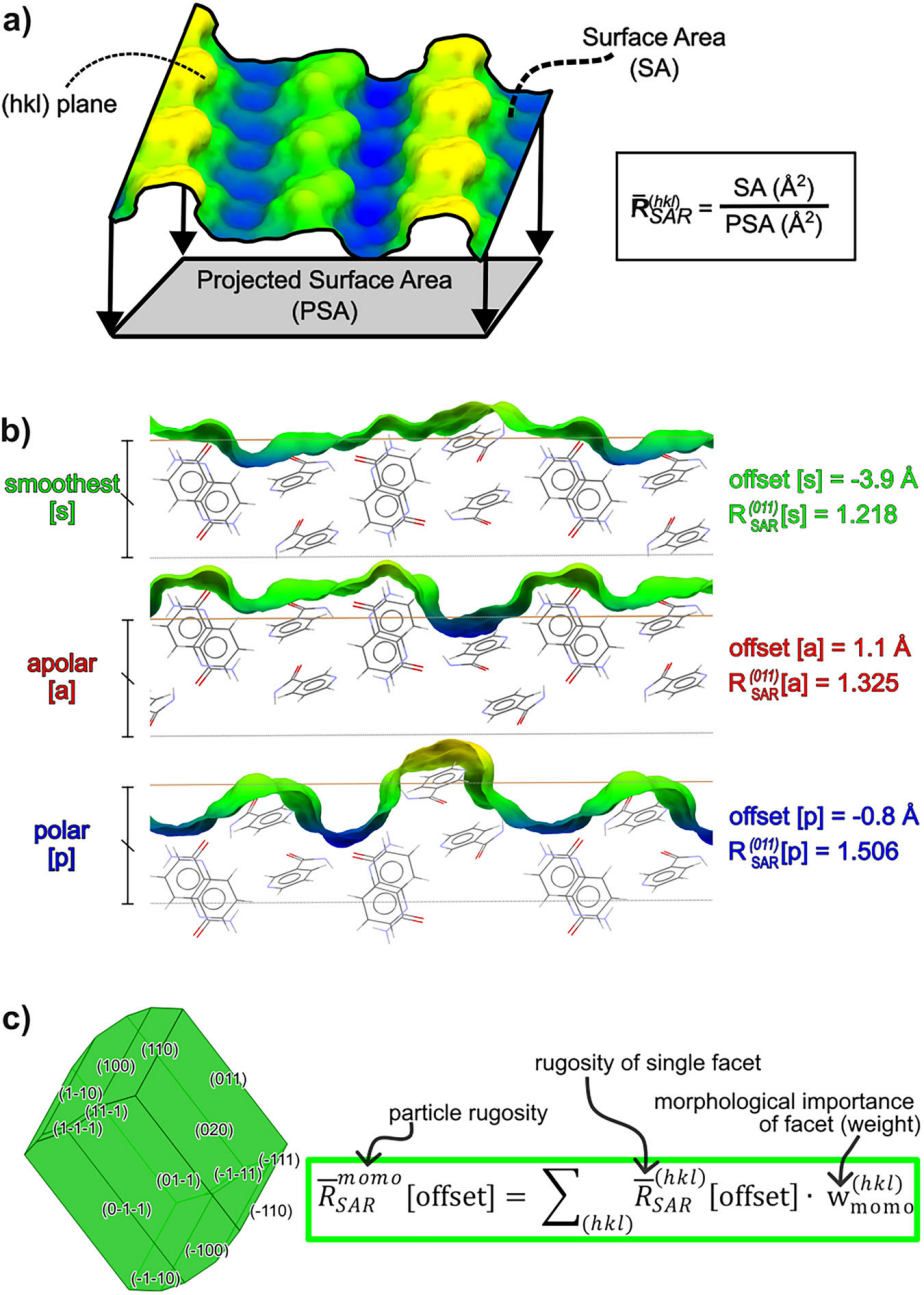
Definitions of rugosity parameters. **a** Graphical representation of surface rugosity descriptor ${\bar{R}}_{{\mbox{SAR}}}^{({{\rm{hkl}}})}$. **b** Depiction of the (011) surface of nicotinamide form-δ (CSD refcode: NICOAM16) cleaved at three distinct offsets. **c** Generated BFDH morphology of nicotinamide form-δ illustrating the calculation of particle rugosity.

In this section, we define and discuss the surface concepts introduced and illustrated in Fig. 1, namely the normalised surface rugosity descriptors for a specific $({{\rm{hkl}}})$ plane, the role of offset in surface cleavage, and the overall particle rugosity parameter.

Surface roughness can be quantified in several ways. The simplest definition is the vertical distance between the highest peak and the lowest trough on a given surface, representing roughness as a single height parameter in Å. Bryant et al.^7^ introduced a related descriptor, ${R}_{{\mbox{depth}}}^{({{\rm{hkl}}})}$, defined as half the peak-to-trough distance or equivalently the distance between a peak and the mid-plane between adjacent packing layers. This metric accounts for interpenetration: negative values indicate surfaces interpenetrating through an average reference plane, while positive values correspond to smooth surfaces separated by a gap. A more informative measure of rugosity is obtained by computing the contact SA of each crystal face and its projected area (PSA) using the surface topology algorithm implemented in CSD-Particle, in which a spherical probe of defined radius is sampled on a regular grid over the surface and the topology is defined by probe intersections with the van der Waals radii of surface atoms, as described by Moldovan et al.^21^.

In both the distance- and area-based metrics, normalisation enables comparison across different systems: depth rugosity is normalised by ${d}_{({hkl})}$, the interplanar spacing, and area rugosity is normalised by the PSA of the surface. In this work, the normalised surface area rugosity (SAR) descriptor^21^ (shown in Fig. 1a as ${\bar{R}}_{{\mbox{SAR}}}^{({{\rm{hkl}}})}$) was chosen. ${\bar{R}}_{{\mbox{SAR}}}^{({{\rm{hkl}}})}=1$ for a perfectly flat surface, increasing with rugosity and remaining strictly positive.

Another critical factor influencing surface topography is the *offset*, defined as the position along the [hkl] direction, expressed in Ångströms, at which the crystal is cleaved to expose the (hkl) face; it is here defined within two interplanar repeat units ${\pm d}_{({hkl})}$. To illustrate its impact, Fig. 1b shows three cleavages of the (011) surface of nicotinamide-δ at offsets of −3.9, −0.8, and 1.1 Å, corresponding to distinct terminations within the same interplanar spacing (${d}_{\left(011\right)}=6.97\mathring{\rm A}$).

We identify three surface cleavage options of interest that generate surface terminations with distinct topological and chemical characteristics: the smoothest surface ([s]), the most apolar surface ([a]), and the most polar surface ([p]). These terminations are obtained by sampling the cleavage offsets along the [hkl] direction, and computing surface descriptors such as the surface rugosity ${\bar{R}}_{{\mbox{SAR}}}^{({{\rm{hkl}}})}$ and the surface density of unsatisfied hydrogen-bond (HB) donors. Smooth facets correspond to the offsets yielding the lowest ${\bar{R}}_{{\mbox{SAR}}}^{({hkl})}$; apolar facets have the lowest density of unsatisfied HB donors; and polar facets have the highest density of unsatisfied HB donors. In this framework, the density of unsatisfied HB donors is used as a physically motivated proxy for surface polarity and potential surface–solvent interactions.

This classification provides a conceptual link to crystallisation behaviour, consistent with established principles in crystal engineering^21,22^: surfaces with low rugosity are expected to pack efficiently and present minimal steric disruption at the crystal–solution interface; apolar terminations, exposing few unsatisfied HB donors, are expected to be stabilised preferentially in apolar solvent environments; and polar terminations, with a high density of unsatisfied HB donors, are expected to interact favourably with polar solvents, consistent with solvent-selection principles in pharmaceutical crystallisation. Solvent effects are therefore implicitly incorporated at the level of surface selection through descriptor-based offset classification rather than explicit solvent modelling.

For clarity throughout the manuscript, rugosity values are abbreviated as $R$, with subscripts indicating the rugosity metric (depth or SAR), a flat hat denoting normalisation (dimensionless), and superscripts specifying the specific (hkl) surface. The abbreviation given in brackets (s, a or p) indicates the offset value used.

Finally, particle rugosity evaluates the overall rugosity of a crystal—it is in fact defined as the sum of individual normalised surface rugosities weighted by the morphological importance of each facet. This concept of particle rugosity was firstly introduced by Montis et al.^13^ but it is generalised here, with the rugosity of each (hkl) surface being the SAR-based one. It is shown in Fig. 1c and expressed in Eq. (1):

1 $${\bar{R}}_{{{\rm{SAR}}}}^{{{\rm{momo}}}}[{{\rm{offset}}}]=\,{\sum }_{({{\rm{hkl}}})}{\bar{R}}_{{{\rm{SAR}}}}^{\left({{\rm{hkl}}}\right)}\left[{{\rm{offset}}}\right]\cdot \,{{{\rm{w}}}}_{{{\rm{momo}}}}^{({{\rm{hkl}}})}$$

where ${{{\rm{w}}}}_{{\mbox{momo}}}^{({{\rm{hkl}}})}$ represents the surface weight derived from the morphology model (momo), which may be experimental (exp) or generated (gen) as computed using either BFDH or the attachment energy models available in the CSD tools (via VisualHabit^23^ using Dreiding^24^, Gavezzotti^25^, Momany^26^, or CLP^27^ potentials).

### Particle rugosity calculations

**Fig. 2:**
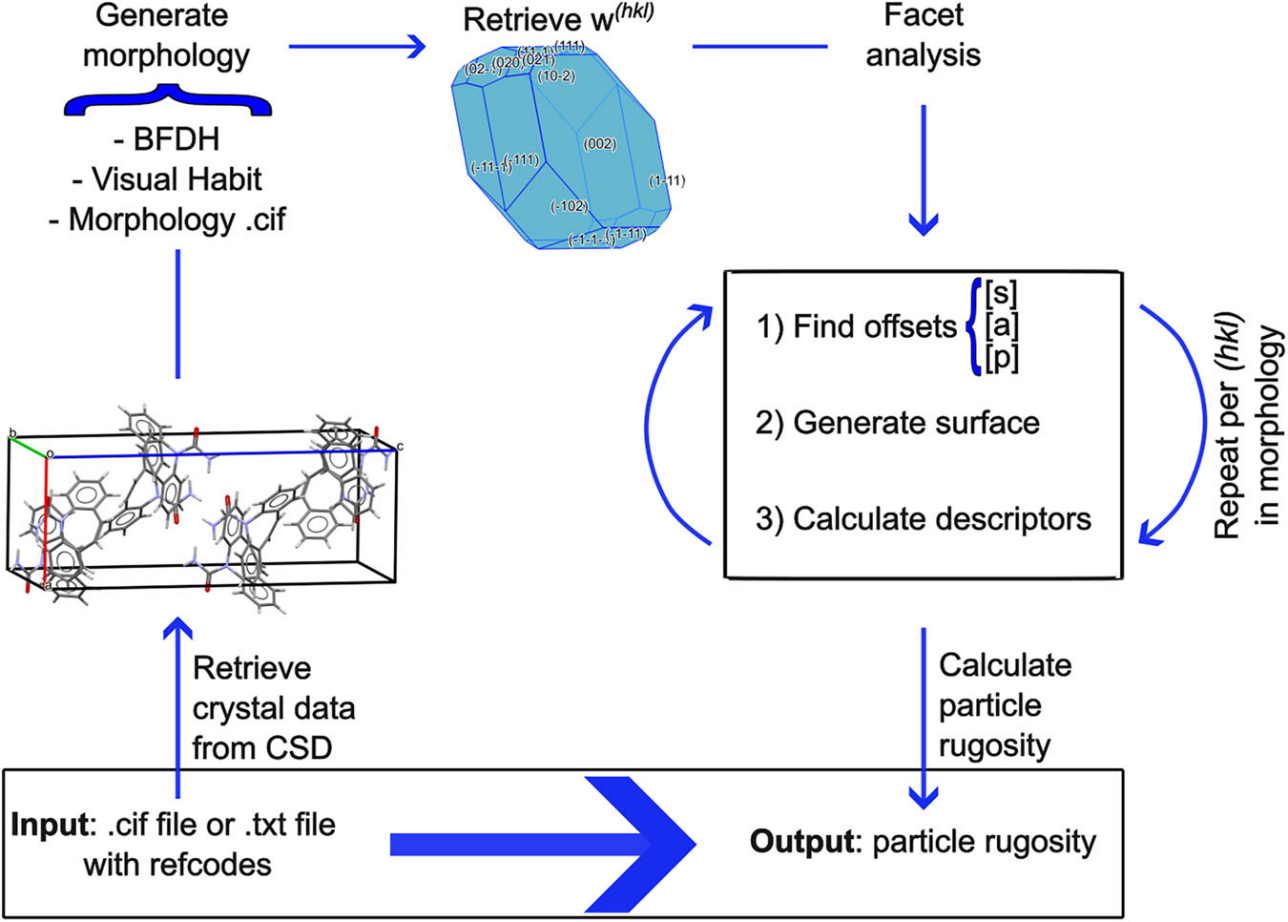
Workflow of the Python algorithm developed to compute particle rugosities. The process begins by loading crystal structure data from a CSD refcode or input file and then generating a morphology using either experimental data or a selected model (BFDH or VisualHabit). Each morphology provides facet weightings, and for each (hkl) facet, the algorithm identifies offsets, generates surface topologies, and calculates surface descriptors, including roughness and hydrogen-bonding properties. These results are combined to compute particle rugosities.

Particle rugosities were computed using a self-made Python algorithm (shown in Fig. 2) which relies on the CSD Python API^28^. The algorithm accepts as input: (i) a crystal structure or CSD refcode list, (ii) a choice of morphology model (momo), and (iii) an integer *n* controlling the resolution of the offset exploration (with an *n* of at least 4 recommended). Morphologies are generated either from experimental data or via CSD-Particle models, which provide facet weightings ${{{\rm{w}}}}_{{\mbox{momo}}}^{({{\rm{hkl}}})}$. Facets are then further analysed using the CSD Surface Analysis^21^ tools.

For each (hkl) facet the analysis involves the generation of surface topologies at different offsets (from -${d}_{({hkl})}$ to ${d}_{({hkl})}$ scanned in steps of ${d}_{({hkl})}/n$), the computation of surface roughness ${\bar{R}}_{{{\rm{SAR}}}}^{\left({{\rm{hkl}}}\right)}$ and the calculation of surface hydrogen-bonding properties (such as HB donor, unsatisfied donor, and acceptor densities). ${\bar{R}}_{{{\rm{SAR}}}}^{\left({{\rm{hkl}}}\right)}$ calculations are performed using the CSD Mercury default sampling values: a probe radius of 1.2 Å and a grid spacing of 0.3 Å. From this ${d}_{({hkl})}$ scan, three specific offsets per facet are identified to represent smoothest ([s]), apolar ([a]), and polar ([p]) cleavage topographies, based on the corresponding surface roughness and hydrogen-bonding characteristics. The values of ${\bar{R}}_{{{\rm{SAR}}}}^{\left({{\rm{hkl}}}\right)}[{{\rm{offset}}}]$ are taken from these identified configurations and used for overall particle rugosity calculations. Since different offsets could yield equivalent terminations, each distinct surface is retained only once, identified by equivalence in its computed descriptors. For each offset, a morphology-weighted particle rugosity ${\bar{R}}_{{{\rm{SAR}}}}^{{{\rm{momo}}}}[{{\rm{offset}}}]$ is calculated. The algorithm currently cannot process disordered structures. When working with these cases, users can manually edit the structure and remove the disorder. In case of input crystal structures with missing H atoms, these are added automatically via the *add_hydrogens(mode* = *“missing”)* built-in method.

The output includes the selected offsets (in Å), facet-level descriptors, and morphology-weighted averages for the particle.

### Datasets

Particle rugosities were computed for six diverse datasets, summarized in Table 1. These datasets were chosen to capture a broad range of crystallization behaviours and structural complexities. Datasets 1–3 include systems with well-characterized experimental challenges, enabling direct correlation between rugosity and crystallization accessibility. Datasets 4 and 5 extend the analysis to general organic molecules and pharmaceutically relevant compounds, ensuring chemical diversity and industrial relevance. Dataset 6 incorporates CSP landscapes, allowing exploration of rugosity trends across hypothetical polymorphs and computationally generated forms. Together, these datasets provide a robust foundation for validating particle rugosity as a potential predictive descriptor.

**Table 1:** Summary of datasets used for the particle rugosity calculations

Dataset number	Dataset name	Description	Number of crystal structures	Reference
1	Nucleation Dataset I	11 Pairs of polymorphic with one elusive polymorph.	22	13
2	Nucleation Dataset II	3 Highly polymorphic systems (> 7): tolfenamic acid, nicotinamide and ROY.	25	18,43,49
3	Growth Dataset	3 Crystal systems with a known crystal growth death zone.	3	8
4	CSD-Organics	Organics subset from the best R-factor list in the CSD.	175 607	20
5	CSD-Drugs	Drug subset from the CSD.	6829	32
6	CSP-Landscapes	6 CSP landscapes from the CPOSS database.	2162	29 + ESI

Crystallographic data for datasets 1–5 were obtained from the CSD, while CSP landscapes were sourced from the CPOSS database that was used to create the CPOSS209 data set^29,30^. Experimental information on crystallization tendencies and physical properties was gathered from the literature (literature information for CSP landscapes can be found in Table S1 of the Supporting Information).

Datasets 4 and 5 were generated using Conquest^31^ (CSD version 5.44 + 3 updates) applying several filtering rules. Searches were restricted to single crystal structures with an R-factor of 5% or lower and containing only common atom types (H/D, C, N, O, S and halogens). Only organic, non-polymeric structures with fully determined 3D coordinates and no errors were retained. The two datasets were generated by applying these search criteria to the “CSD best R-factor” and “CSD-drug”^32^ subset lists already available in Conquest.

Particle rugosity calculations used an offset resolution number *n* = 25 for datasets 1–3 to maximize accuracy, and *n* = 4 for larger datasets (4–6) for computational efficiency. All computations were performed on the Hamilton HPC cluster at Durham University.

### Rugosity and molecular size correlation

The number of atoms per molecule for all structures in datasets were calculated using the CSD Python API^28^ and analysed in relation to particle rugosities. For multi-component structures, only the component with the highest atom count was considered. Dataset 4 data was binned by number of atoms and for each bin, the median rugosity value and deviation across its distribution was calculated. These median values as a function of number of atoms were then fitted to various functional forms (linear, logarithmic, exponential, and quadratic polynomial) using nonlinear least squares implemented in an in‑house Python script built on NumPy^33^ and SciPy^34^.

## Results

### Variable selection for particle rugosity calculations

**Fig. 3:**
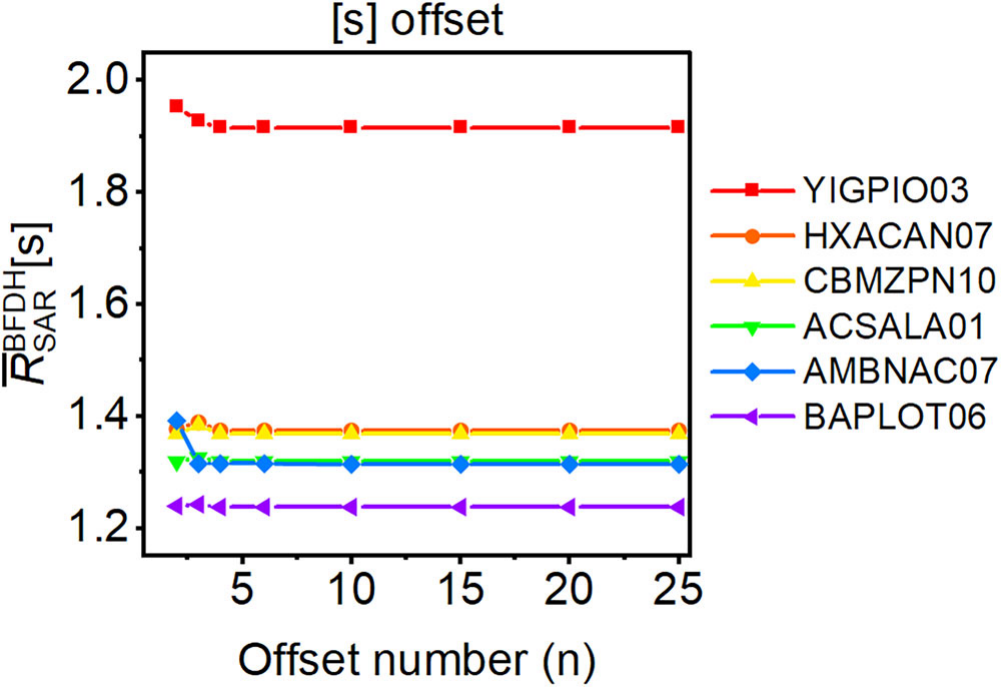
Influence of the offset resolution *n* on particle rugosity for the [s] offset data. Results are reported for six representative systems, identified by their CSD refcodes in the legend at the right of the figure. All calculations were performed using the BFDH morphology model.

Our calculations of particle rugosity depend on two user-defined parameters: the offset resolution number $n$ and the morphology model (momo). In this section, we assess the influence of these parameters on the calculated particle rugosities and justify the choices adopted for the remainder of the analyses. The six crystal structures selected for this assessment exercise (with refcodes in Fig. 3) correspond to a varied set of compounds: from small rigid molecules like theophylline (BAPLOT06) to large and flexible molecules like ritonavir (YIGPIO03).

Figure 3 illustrates the effect of varying the offset resolution $n$ on the calculated particle rugosity with the smoothest offset criteria [s], with similar plots for [a] and [p] given in the Supporting Information (Fig. S1). For consistency, all calculations in this analysis were performed using the BFDH momo. Increasing $n$ enhances the surface sampling resolution, as $2n$ effectively corresponds to the number of distinct surface cuts explored. As a result, the calculated particle rugosity is expected to converge with increasing $n$. As shown in Fig. 3, convergence is achieved for all systems at $n\ge 4$ - hence, $n$ will be set to 4 for rugosity calculations on larger datasets of structures (datasets 4–6). For exhaustive sampling in smaller subsets (datasets 1–3), we used $n=25$.

We next examined the effect of the morphology model on the resulting particle rugosities. Five available models—BFDH and VisualHabit (VH) with four different force-fields (CLP, Dreiding, Gavezzotti and Momany)—were tested for our selected six structures and an overall average rugosity across all models was calculated (${\bar{R}}_{{{\rm{SAR}}}}^{{{\rm{average}}}}[{{\rm{s}}}]$). Table 2 reports all the particle rugosity values for the six representative systems together with an average deviation from the

**Table 2:** Comparison of particle rugosity values across the five available morphology models (BFDH and VisualHabit with multiple force-field potentials)

Compound-Form	Refcode	${\bar{{{\rm{R}}}}}_{{{\rm{SAR}}}}^{{{\rm{average}}}}[{{\rm{s}}}]$	${\bar{{{\rm{R}}}}}_{{{\rm{SAR}}}}^{{{\rm{BFDH}}}}[{{\rm{s}}}]$	${\bar{{{\rm{R}}}}}_{{{\rm{SAR}}}}^{{{\rm{CLP}}}}[{{\rm{s}}}]$	${\bar{{{\rm{R}}}}}_{{{\rm{SAR}}}}^{{{\rm{Dreiding}}}}[{{\rm{s}}}]$	${\bar{{{\rm{R}}}}}_{{{\rm{SAR}}}}^{{{\rm{Gavezzotti}}}}[{{\rm{s}}}]$	${\bar{{{\rm{R}}}}}_{{{\rm{SAR}}}}^{{{\rm{Momany}}}}[{{\rm{s}}}]$
pABA—α	AMBNAC07	1.286	1.314	1.279	1.281	1.280	1.278
Aspirin—I	ACSALA01	1.317	1.320	1.321	1.288	1.323	1.331
Paracetamol—I	HXACAN07	1.350	1.375	1.338	1.348	1.345	1.346
Carbamazepine—III	CBMZPN10	1.359	1.369	1.353	1.395	1.350	1.328
Theophylline—II	BAPLOT06	1.230	1.238	1.234	1.228	1.224	1.228
Ritonavir—II	YIGPIO03	1.829	1.915	1.801	1.805	1.802	1.821
Average deviation from${\bar{{{\rm{R}}}}}_{{{\rm{SAR}}}}^{{{\rm{average}}}}[{{\rm{s}}}]$			0.027	0.010	0.016	0.010	0.011
Time^a^ elapsed per structure (s), *n* = 4			37 ± 11	59 ± 22	70 ± 29	56 ± 30	78 ± 36
Time^a^ elapsed per structure (s), *n* = 25			174 ± 37	282 ± 83	310 ± 119	290 ± 145	296 ± 162

For each structure, rugosity values obtained using the different morphology models were averaged (${\bar{{{\boldsymbol{R}}}}}_{{{\bf{SAR}}}}^{{{\bf{average}}}}{{\boldsymbol{[}}}{{\bf{s}}}{{\boldsymbol{]}}}$), and the average deviation from ${\bar{{{\boldsymbol{R}}}}}_{{{\bf{SAR}}}}^{{{\bf{average}}}}{{\boldsymbol{[}}}{{\bf{s}}}{{\boldsymbol{]}}}$ for each momo reported. The computational time per structure (in *s*) is reported in the last two rows for two offset resolutions, $n=4$ and $n=25$.

^a^Time of calculation on a single core of a 13th Gen Intel® Core™ i5-1335U processor and 16 GB of RAM.

${\bar{R}}_{{{\rm{SAR}}}}^{{{\rm{average}}}}[{{\rm{s}}}]$ per model. Overall, all VH calculations are similar to each other with only small variations in the particle rugosities. The BFDH particle rugosities are also very similar to the VH with some systems (AMNAC07 and YIGPIO03) showing slightly larger differences. Whilst the differences in the computed particle rugosities across momos are small, the computational time required is notably less demanding for the BFDH momo than for the energy based models. Since we are studying large sets of structures here, we decided to proceed with the BFDH models for the large analyses. However, all five momos are implemented in the algorithm and accessible through our code.

### Application to dataset-1: difficult vs easy crystallisation in 11 pairs of polymorphs

As an initial validation and application of SAR-based particle rugosity calculations, we studied Dataset 1, which comprises 11 pairs of polymorphs: one form categorized as “easy” to crystallize and another as “difficult.” This dataset was originally compiled by Montis et al.^13^, who calculated particle rugosities using the simpler 1D depth descriptor (${\bar{R}}_{{{\rm{depth}}}}^{{{\rm{BFDH}}}}[{{\rm{s}}}]$).

Our newly computed SAR-based particle rugosities (${\bar{R}}_{{{\rm{SAR}}}}^{{{\rm{BFDH}}}}[{{\rm{s}}}]$) are presented in Table 3, alongside Montis’ original values, both calculated using the smoothest offset topology. The classification of polymorphs into “easy” and “difficult” was based on Montis’ detailed analysis of crystallization conditions. For example, 5-Br-aspirin form I (easy) can be grown by slow evaporation of concentrated acetonitrile solutions^35^, whereas form II (difficult) crystallizes only in the presence of an impurity (its corresponding anhydride), which inhibits the rapid II → I transformation^36^. Similarly, carbamazepine form III (easy) is readily obtained from ethanol^37^, while single crystals of the catemeric form V (difficult) require vapor-phase templating on a dihydrocarbamazepine surface^38^.

**Table 3:** Comparison of particle rugosities obtained using two descriptors (${\bar{R}}_{{\mbox{depth}}}^{{\mbox{BFDH}}}[{{\rm{s}}}]$ and ${\bar{R}}_{{\mbox{SAR}}}^{{\mbox{BFDH}}}[{{\rm{s}}}]$) for 11 polymorphic systems, each with an “easy” and a “difficult” form

Compound—difficult/easy forms	Difficult crystallisation			Easy crystallisation			Difficult—Easy	
	Refcode	$-{\bar{{{\rm{R}}}}}_{{{\rm{depth}}}}^{{{\rm{BFDH}}}}[{{\rm{s}}}]$	${\bar{{{\rm{R}}}}}_{{{\rm{SAR}}}}^{{{\rm{BFDH}}}}[{{\rm{s}}}]$	Refcode	$-{\bar{{{\rm{R}}}}}_{{{\rm{depth}}}}^{{{\rm{BFDH}}}}[{{\rm{s}}}]$	${\bar{{{\rm{R}}}}}_{{{\rm{SAR}}}}^{{{\rm{BFDH}}}}[{{\rm{s}}}]$	$-\triangle {\bar{{{\rm{R}}}}}_{{{\rm{depth}}}}^{{{\rm{BFDH}}}}[{{\rm{s}}}]$	$\triangle {\bar{{{\rm{R}}}}}_{{{\rm{SAR}}}}^{{{\rm{BFDH}}}}[{{\rm{s}}}]$
pABA—β/α	AMBNAC08	0.091	1.324	AMBNAC07	0.061	1.314	0.030	0.010
5-BrAspirin—II/I	NUWTIJ01	0.117	1.510	NUWTIJ	0.061	1.337	0.057	0.173
Aspirin—II/I	ACSALA15	0.121	1.346	ACSALA01	0.070	1.320	0.051	0.026
Carbamazepine—V/III	CBMZPN16	0.193	1.421	CBMZPN10	0.125	1.369	0.068	0.051
Paracetamol—II/I	HXACAN08	0.216	1.591	HXACAN07	0.153	1.375	0.064	0.216
Curcumin—II/I	BINMEQ08	0.289	2.067	BINMEQ	0.233	1.968	0.056	0.099
Dapsone—V/III	DAPSUO18	0.106	1.308	DAPSUO14	0.110	1.340	−0.004	−0.032
Theophylline—IV/II	BAPLOT03	0.161	1.491	BAPLOT06	0.025	1.238	0.137	0.253
Axitinib—XLI/XXV	VUSDIX04	0.170	1.523	VUSDIX	0.115	1.414	0.055	0.109
Ritonavir—II/I	YIGPIO03	0.265	1.915	YIGPIO02	0.114	1.693	0.151	0.237
Rotigotine—II/I	RALMOG01	0.372	1.686	RALMOG	0.402	1.909	−0.030	−0.224

For systems in the top half of the table, the polymorph of easy crystallisation is also the most stable. For systems in the bottom half of the table, the polymorph of difficult crystallisation is also the most stable.

The results strongly support the observation by Montis et al. that polymorphs which crystallize more easily exhibit lower particle rugosities. The updated metric ${\bar{R}}_{{{\rm{SAR}}}}^{{{\rm{BFDH}}}}[{{\rm{s}}}]$ shows consistent trends with the previously reported ${\bar{R}}_{{{\rm{depth}}}}^{{{\rm{BFDH}}}}[{{\rm{s}}}]$: in nearly all cases, “easy” forms display lower rugosity values than their “difficult” counterparts (positive difficult–easy difference), except for Rotigotine and Dapsone, which deviate from this trend for both metrics. These exceptions suggest that crystallization kinetics for these systems are influenced by additional factors beyond surface roughness with molecular flexibility being another key characteristic^39,40^.

Overall, the strong agreement between the two metrics reinforces the validity of using SAR-based rugosity as a potential descriptor of crystallization behaviour. In particular, ${\bar{R}}_{{{\rm{SAR}}}}^{{{\rm{BFDH}}}}$ captures the full three-dimensional surface topography and chemically distinct terminations exposed at different cleavage offsets, and this feature enables qualitative surface classification and offset selection that are not accessible using ${\bar{R}}_{{{\rm{depth}}}}^{{{\rm{BFDH}}}}$. This added descriptive capability, combined with automation, makes ${\bar{R}}_{{{\rm{SAR}}}}^{{{\rm{BFDH}}}}$ particularly suited for systematic analyses of surface diversity across broader datasets.

### Application to dataset-2: highly polymorphic systems

**Fig. 4:**
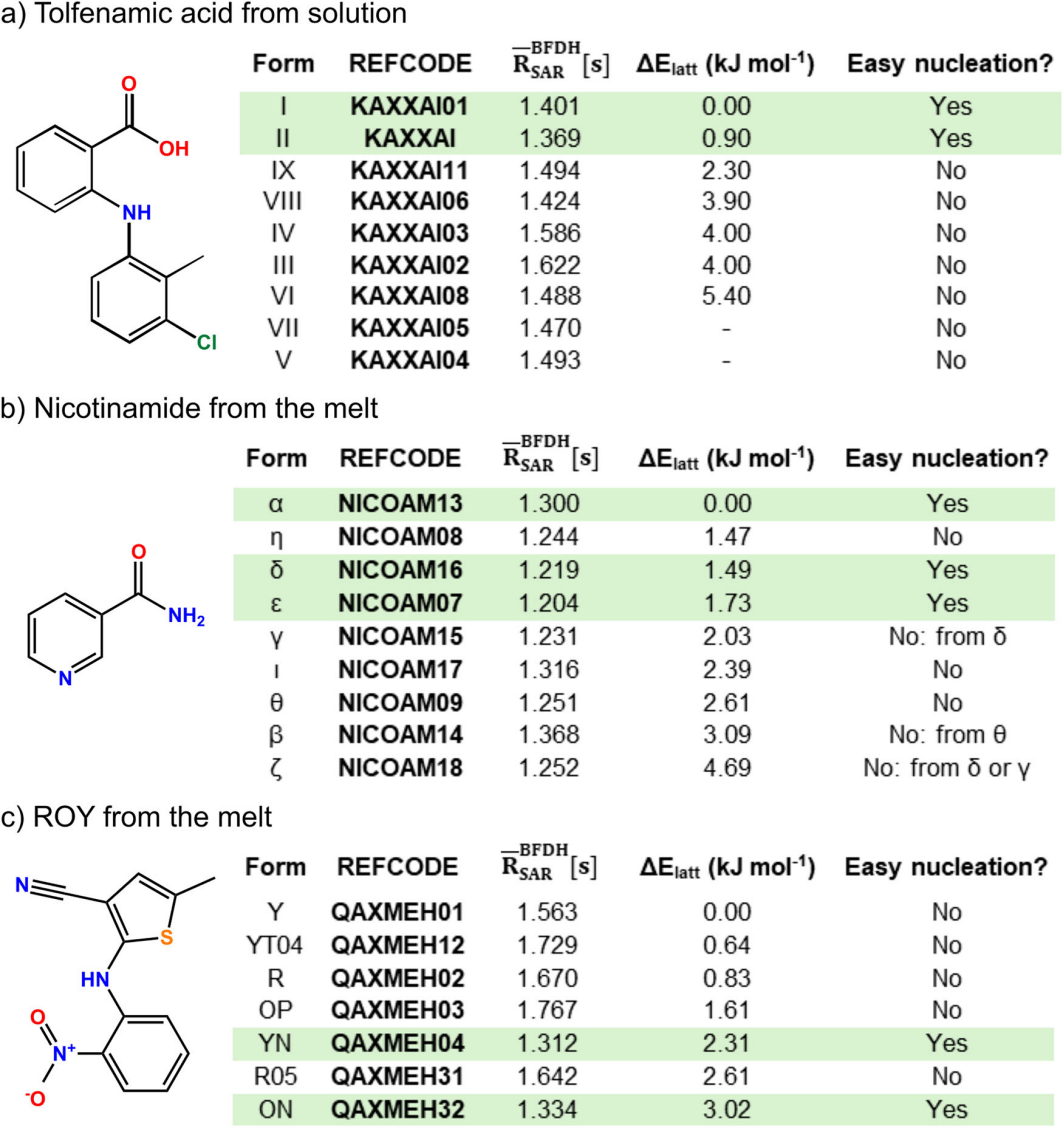
Particle rugosities and polymorphism. Particle rugosity data for polymorphic forms of **a** **tolfenamic acid** (relative lattice energies from ref. ^18^, PBE-TS + MBD method), **b** **nicotinamide** (relative lattice energies from ref. ^43^, optPBE-vdw method), and **c** **ROY** (relative lattice energy from ref. ^58^, B86bPBE-XDM + SCS-MP2D method; self-nucleation data from ref. ^49^). Rugosities were computed using the smoothest offset [s] with $n=25$ offsets. Polymorphs are listed in order of increasing relative lattice energy ($\Delta {E}_{{\mbox{latt}}}$).

We next calculated particle rugosities for three highly polymorphic compounds with well-documented crystallization conditions: tolfenamic acid, nicotinamide, and ROY. For consistency, the smoothest offset cleavage criteria was used for surface generation across all systems, as this best reflects conditions for melt crystallisation which are analysed in depth here. Figure 4 summarizes the results for each system, including chemical structures, relative lattice energies (from prior literature), and CSD refcodes. Most polymorphs lie within 4 kJ·mol^−1^ ( ≈ 1 kcal·mol^−1^) of the most stable form, indicating that all are energetically accessible.

Tolfenamic acid, an anti-inflammatory drug, exhibits nine polymorphs, with the ninth only recently discovered^18^. Polymorphs I and II are the most thermodynamically stable and readily crystallize from a wide range of solvents and conditions. In contrast, the remaining forms are significantly harder to obtain, requiring specialized techniques such as sublimation onto polymer substrates, solid solution preparation, or slow evaporation in the presence of impurities. Rugosity calculations reveal that forms I and II have the smoothest surfaces (Fig. 4a). Their low rugosity values align with their ease of crystallization and thermodynamic stability, suggesting that smoother surfaces facilitate nucleation kinetics. Notably, within this system, particle rugosity and lattice energy show a concordant trend, with higher-energy polymorphs tending to exhibit rougher surfaces and greater difficulty in crystallization. However, this behaviour is system-dependent, and, as shown in Fig. 4, rugosity does not exhibit a universal correlation with lattice energy across all compounds, instead providing complementary insight into crystallization behaviour.

Nicotinamide (vitamin B3) has nine polymorphs. Form α was discovered in 1954^41^, while form β appeared 57 years later^42^. A recent comprehensive study^43^ reported seven additional polymorphs obtained via melt crystallization. Interestingly, some polymorphs nucleate spontaneously from the melt, while others require templating on the surfaces of different polymorphs. Our analysis (Fig. 4b) shows that forms requiring cross-nucleation—β (from θ), γ (from δ), and ζ (from δ or γ)—all exhibit higher rugosity than their respective template forms. For example, β (${\bar{R}}_{{\mbox{SAR}}}^{{\mbox{BFDH}}}[{{\rm{s}}}]=1.368$) nucleates only after θ ($1.251$), and γ ($1.231$) requires δ ($1.219$) to nucleate. This trend suggests that smoother surfaces promote homogeneous nucleation, while rougher surfaces necessitate heterogeneous nucleation via templating. The commercially available α form, despite being thermodynamically favoured, has one of the highest rugosity values ($1.300$), highlighting that thermodynamic stability can compensate for kinetic disadvantages. Similarly, form η has a low lattice energy (it is thermodynamically favoured), but a slightly higher rugosity relative to forms δ, ε, and γ and in fact it has been reported to nucleate spontaneously from the melt, albeit with extremely low probability (growth is kinetically hindered).

ROY (Red, Orange, Yellow), a synthetic intermediate in olanzapine production, is the most polymorphic compound known, with 14 experimental forms^44–48^. Melt crystallization studies^49^, which allow access for the seven polymorphs (shown in Fig. 4c) show that only polymorphs ON and YN self-nucleate, while others require templating. Our rugosity analysis reveals that ON and YN have the lowest rugosity values (ON: 1.334, YN: 1.312), whereas cross-nucleating forms range from 1.563 to 1.767. This stark difference reinforces that smoother surfaces correlate with spontaneous nucleation. Interestingly, rugosity appears more predictive than lattice energy: form Y, the most stable polymorph, has a high rugosity (1.563) and cannot self-nucleate, while ON and YN, despite being less stable (2.3 and 3.0 kJ·mol^−1^ above Y), nucleate readily. These findings highlight that kinetic factors linked to surface topology can override thermodynamic driving forces, revealing the complementary roles of rugosity and lattice energy in predicting crystallization behaviour.

### Application to dataset-3: crystal growth dead-zone

In the previous sections, we examined the role of rugosity in overall crystallization and nucleation. Here, we investigate its impact on crystal growth by analysing systems that exhibit a growth dead-zone-facets that fail to grow at low supersaturation despite the existence of thermodynamic driving force.

**Fig. 5:**
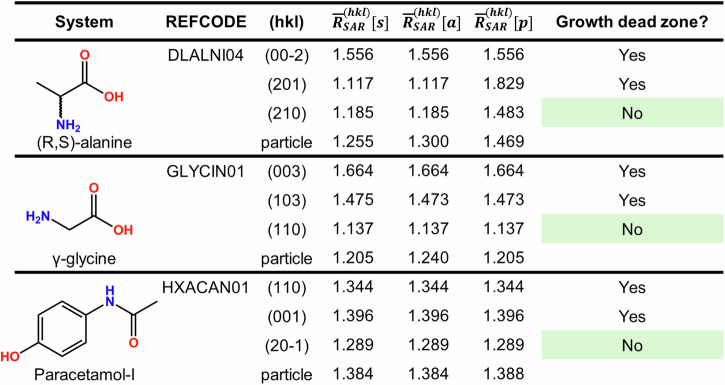
Surface rugosity analysis for crystal facets of systems exhibiting growth dead-zones (ref. ^8^). Facet-specific rugosity values (${\bar{R}}_{{\mbox{SAR}}}^{\left({{\rm{hkl}}}\right)}$) are shown for the three offset criteria (see section “Methods” and Fig. 1) alongside whole-particle rugosity (${\bar{R}}_{{\mbox{SAR}}}^{{\mbox{BFDH}}}$).

We revisited the systems reported by Liu et al.^8^—*(R,S)*-alanine, γ-glycine, and paracetamol form I—and computed SAR rugosities for several facets, some of which known to exhibit crystal growth dead-zones (Fig. 5). We report the rugosity calculations at all three offset criteria, but since these dead-zones were reported from growth experiments in aqueous solutions, the polar offset is the most representative of the reported growth conditions.

Consistent with prior observations, facets exhibiting growth dead-zones show significantly higher structural rugosity compared to facets without dead-zones. For γ-glycine, the dead-zone faces (00-1) and (103) exhibit rugosity values of 1.664 and 1.473, compared to 1.137 for the no dead-zone (110) face. Similarly, for paracetamol form I the (110) and (001) faces both have growth dead-zones and elevated rugosity values of 1.344 and 1.396, respectively, while the (20-1) face has no dead-zone and a lower rugosity of 1.289. While for these two systems the offset criterion does not have an impact on rugosity values, it matters for *(R,S)*-alanine instead. For the system, it is the polar offset that follows the previously observed trend: the dead-zone facets (00-1) and (201) have ${\bar{R}}_{{\mbox{SAR}}}^{\left({{\rm{hkl}}}\right)}[{{\rm{p}}}]$ values of 1.556 and 1.829, respectively, substantially higher than the non-dead-zone (210) face (${\bar{R}}_{{\mbox{SAR}}}^{\left({{\rm{hkl}}}\right)}[{{\rm{p}}}]$ = 1.483). Furthermore, for alanine and γ-glycine, the polar rugosities of dead-zone faces are significantly higher than the overall particle rugosity (${\bar{R}}_{{\mbox{SAR}}}^{\left({{\rm{hkl}}}\right)}[{{\rm{p}}}]$ = 1.469 and 1.205, respectively), indicating that these facets present particularly challenging growth environments.

These findings are consistent with the hypothesis that, at low supersaturation, growth on highly corrugated surfaces is kinetically hindered by the energy barrier associated with displacing tightly bound solvent molecules before incorporating new growth units. The observed separation in ${\bar{R}}_{{\mbox{SAR}}}^{\left({{\rm{hkl}}}\right)}$ values between dead-zone and non-dead-zone faces suggests that surface rugosity may serve as a useful descriptor of both bulk nucleation propensity and face-specific growth kinetics, although further validation across a wider range of systems would be required to establish its predictive capability. Additionally, applying the polar offset criterion for surface cleavage appears to enhance differences in rugosity and aids in rationalising crystallisation behaviour, as illustrated for the DLALNI04 system, but its general applicability across diverse solvents remains to be further assessed.

Within individual structures or polymorphic families, rugosity provides a useful descriptor for rationalising differences in growth behaviour between facets. However, its interpretation across structurally distinct systems requires caution, as absolute rugosity values depend on system-specific characteristics. Overall, these results highlight the relevance of rugosity as a framework for analysing crystal growth processes, while also underlining the need for further validation to establish its broader predictive applicability.

### Application to datasets 4 and 5: rugosity trends in molecular crystals

**Fig. 6:**
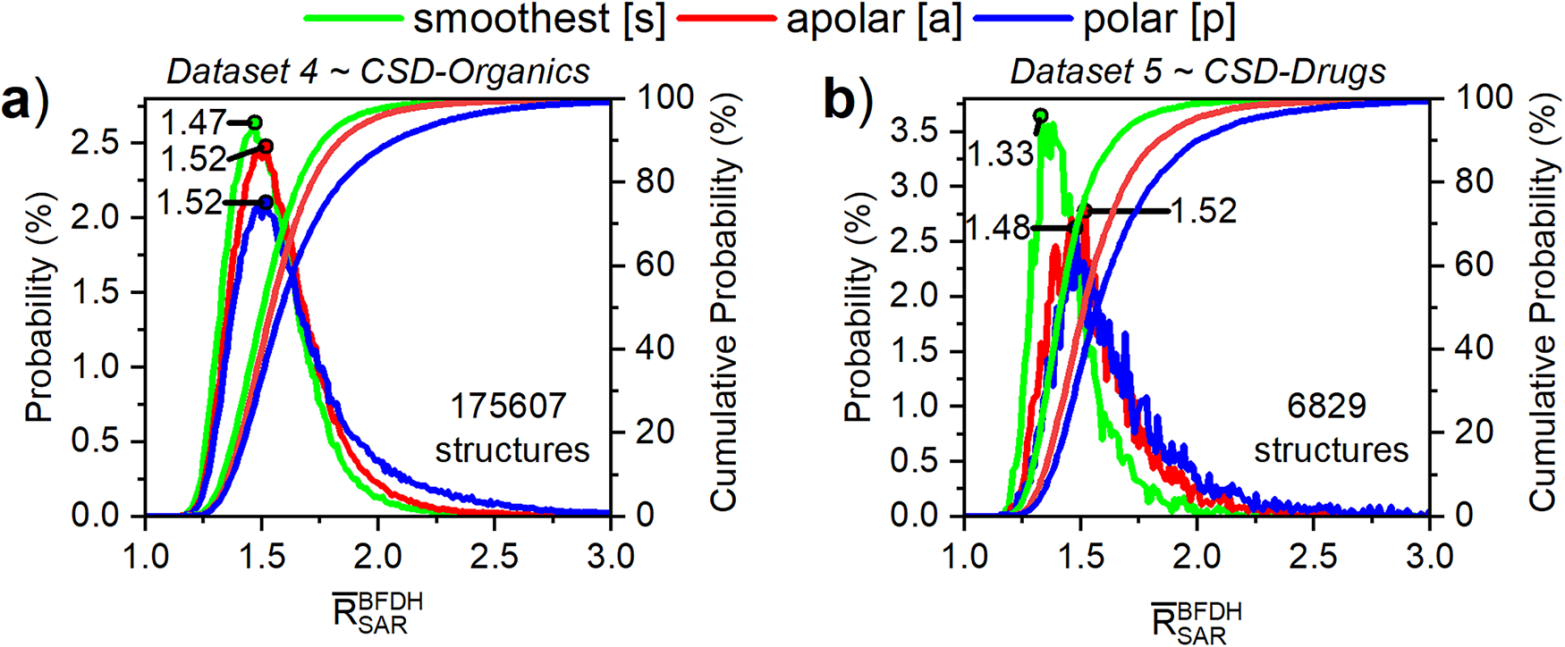
Distribution of particle rugosity $({\bar{R}}_{{{\rm{SAR}}}}^{{{\rm{BFDH}}}})$ for two CSD subsets. **a** Dataset 4 (CSD-organics) and **b** Dataset 5 (CSD-drugs). Rugosities were computed for morphologies generated under three offset criteria: smoothest (lowest ${\bar{R}}_{{\mbox{SAR}}}^{\left({{\rm{hkl}}}\right)}$), apolar (lowest density of hydrogen-bond donors), and polar (highest density of hydrogen-bond donors). Cumulative probability curves highlight broader rugosity ranges for polar morphologies compared to smoothest and apolar offsets.

We analysed particle rugosity distributions for two large subsets of molecular crystals from the CSD: (a) CSD-organics (175,607 structures) and (b) CSD-drugs (6829 structures). Details of subset generation are provided in the “Methods” section. The aim was to characterize typical rugosity values for experimentally observed molecular crystals and drug-like compounds. Distributions for both datasets, calculated using the three offset cleavage criteria are shown in Fig. 6.

For the smoothest offset [s], the CSD-organics dataset exhibits a modal rugosity between 1.46 and 1.47 (2.64% of entries), whereas the drug subset peaks at 1.33, indicating that crystal structures of approved drug molecules tend to form smoother particle surfaces. Overall, 80% of CSD-organics structures have ${\bar{R}}_{{\mbox{SAR}}}^{{\mbox{BFDH}}}[{{\rm{s}}}]$ values within 1.16–1.66, while this range narrows to 1.16–1.54 for drug-like molecules.

For the CSD-organics, the [s] distribution of rugosities is very similar to the [a] and [p] ones because nearly half (49%) of CSD-organics structures lack HB donors, making the data calculated with the [s], [a], and [p] criteria converge. To address this bias, we performed an additional analysis of the CSD-organics by filtering for structures containing at least one H-bond donor (see Supporting Information, Fig. S2). For this CSD-organics-HBonds subset, the modal ${\bar{R}}_{{\mbox{SAR}}}^{{\mbox{BFDH}}}[{{\rm{p}}}]$ shifted higher to 1.56. The drug subset—where 94% of structures contain H-bond donors—is not biased by this, with the [a] and [p] distributions being shifted to higher rugosities than the [s] distribution.

Despite similar modal values for [a] and [p] distributions in both subsets, morphologies generated under the polar growth assumption consistently exhibited rougher surfaces than those generated with the apolar model, as shown by the cumulative probability curves in Fig. 6. Specifically, 80% of polar morphologies span wider rugosity intervals than apolar and smoothest counterparts (1.17–1.85 for CSD-organics and 1.16–1.82 for drugs), reflecting the influence of solvent polarity on surface termination and topological complexity.

**Fig. 7:**
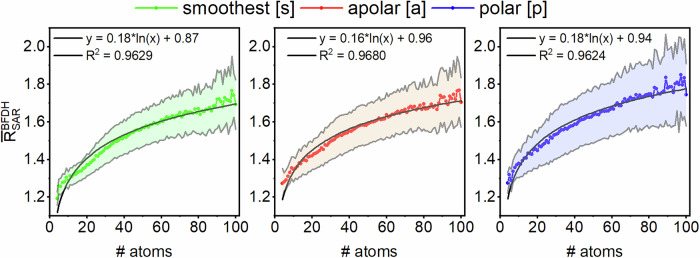
Correlation between particle rugosity $({\bar{R}}_{{{\rm{SAR}}}}^{{{\rm{BFDH}}}})$ and molecular size (number of atoms per molecule) for structures in Dataset 4. Each point represents the median rugosity for molecules of a given size. Shaded bands indicate ± the median absolute deviation (MAD), illustrating the spread of rugosity values within each molecular size bin. Logarithmic fitting curves for the median values are shown in black, with corresponding equations and ${R}^{2}$ values for each offset. Logarithmic fitting curves for the MAD values are show in their offset colour, with corresponding upper and lower equations. Larger molecules exhibit higher rugosity, reflecting increased structural complexity.

As an additional analysis, we examined the relationship between molecular size and particle rugosity for all structures in the CSD-organics dataset. Figure 7 shows rugosity distributions at the three offsets for each molecular size, along with fitted logarithmic trends, which provided the best fit. Larger molecules exhibit higher particle rugosities, consistent with their intrinsically increased conformational flexibility and complex surface topographies. In addition, the dispersion of rugosity values increases with molecular size, as reflected by the widening median absolute deviation (MAD) bands, indicating greater variability in particle morphology for larger molecules. The analysis was performed also on single-component structures only from the CSD-organics dataset (see Fig. S3 in the Supporting Information), showing a very similar trend. This correlation, together with rugosity benchmarks from CSD subsets, provides practical thresholds for crystal structure prediction, and helps identify predicted forms most likely to be experimentally accessible. In a pharmaceutical context, such size-dependent rugosity benchmarks could support early-stage solid-form screening by risk-assessing candidate crystal structures with specific surface properties, thus guiding the selection of crystal forms for subsequent manufacturing and crystallisation development.

### Application to dataset-6: crystal structure prediction

**Fig. 8:**
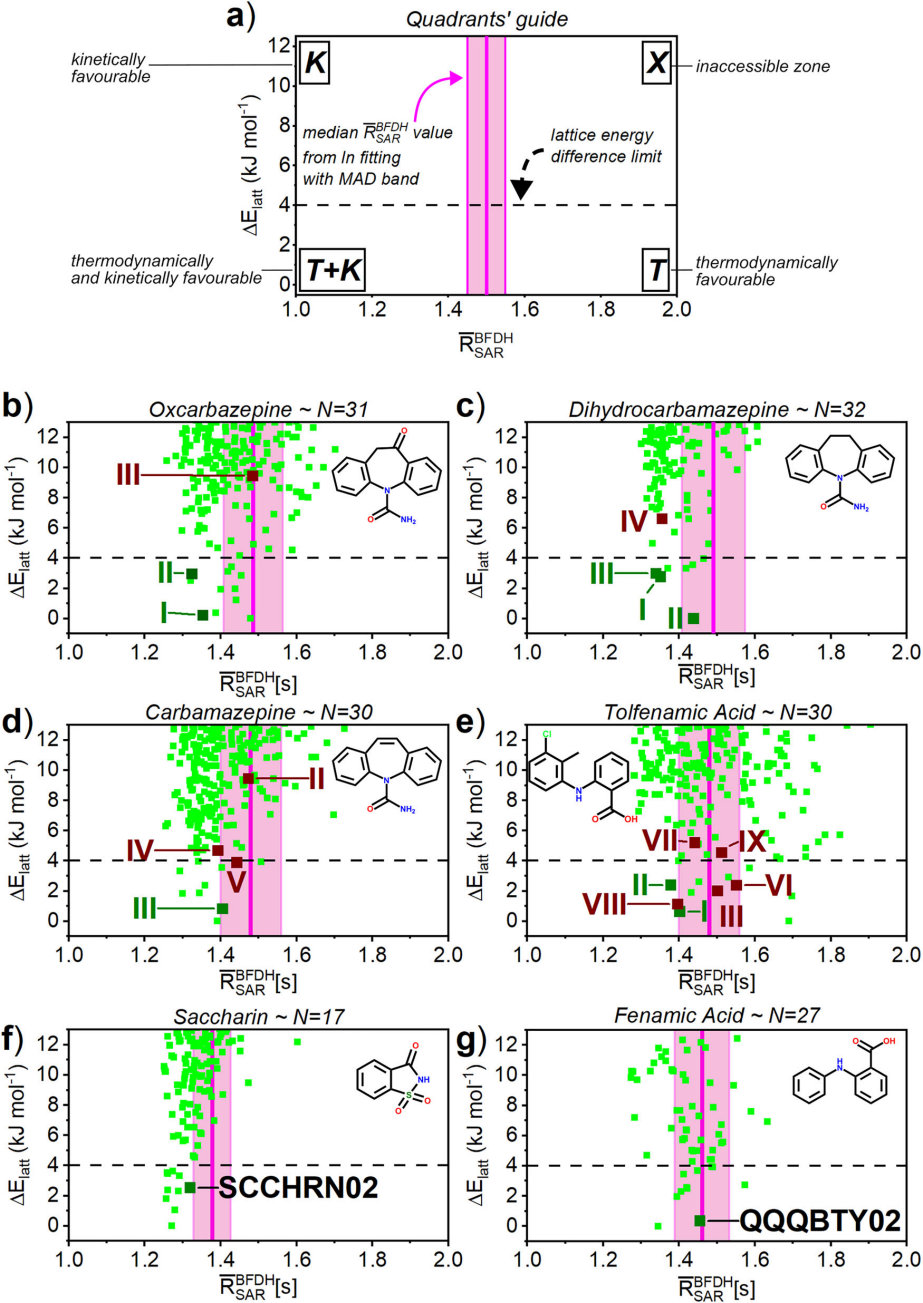
Particle rugosity as a probe for CSP landscape analysis. **a** Guide to analyse the energy vs rugosity CSP landscapes, with the names of each quadrant. CSP landscapes of four polymorphic structures—**b** oxcarbazepine, **c** dihydrocarbamazepine, **d** carbamazepine and **e** tolfenamic acid and of two monomorphic structures—**f** saccharin, and **g** fenamic acid. For the polymorphic structures, the experimentally easy to access structures are labelled in dark green, while the difficult to crystallise ones are in red. The .cif files of the CSP generated structures and lattice energy data were provided by Prof. S. L. Price from the CPOSS database. For each system, the number of atoms per molecule *N* and their molecular structure are shown.

In this section, we extend the application of particle rugosity to the analysis of CSP landscapes. Six CSP landscapes from the CPOSS database were examined, comprising four polymorphic systems and two monomorphic systems (Fig. 8; see the Table S1 in the Supporting Information for literature details). For each system, the CSP landscape is represented as a plot of relative lattice energy (${\Delta {{\rm{E}}}}_{{{\rm{latt}}}}$) versus particle rugosity, calculated using the smoothest ([s]) offset condition and the BFDH morphology. Experimentally observed crystal structures are highlighted in dark green or red according to their reported crystallisation propensity, with green indicating easy crystallisation and red indicating difficult crystallisation. Figure S4 in the Supporting Information shows the predicted landscapes for the six systems at the apolar [a] and polar [p] offset as well.

To rationalise these landscapes, the data were partitioned into four quadrants using two reference boundaries (Fig. 8a). The first boundary is thermodynamic and corresponds to a ${\Delta {{\rm{E}}}}_{{{\rm{latt}}}}$ threshold of approximately 4 kJ mol^−1^ ( ≈ 1 kcal mol^−1^). This value reflects the well-established observation that nearly 90% of polymorph pairs differ in lattice energy by less than 1 kcal mol^−1^, and is also comparable to the typical uncertainty associated with computed lattice energies^39,50–55^. The second boundary reflects kinetic considerations and is shown as a pink vertical band. It is constructed from the analysis of the distribution of particle rugosities for molecules of similar size in the CSD, it shows the mean rugosity value and its MAD (see Fig. 7), and provides a reference for typical experimentally accessible surface roughness. We emphasise that these two boundaries are empirical, the kinetic one is also system-dependent, and both are intended as a qualitative guide rather than a strict predictive threshold.

Based on these two boundaries, four regions of the CSP landscape are defined. The bottom-left quadrant (“T + K”) contains structures that are both thermodynamically favourable (low ${\Delta {{\rm{E}}}}_{{{\rm{latt}}}}$) and kinetically accessible (low rugosity). The bottom-right quadrant (“T”) contains structures favoured thermodynamically but disfavoured kinetically due to high rugosity. The top-left quadrant (“K”) includes structures that are kinetically favourable (relatively lower rugosity) but thermodynamically less stable. Finally, the top-right quadrant (“X”) corresponds to structures that are neither thermodynamically nor kinetically favoured. Figure 8a summarises these definitions and provides a guide for interpreting the individual landscapes shown in Fig. 8b–g.

This quadrant-based analysis reveals several clear trends. Across all six systems examined, experimental polymorphs that crystallise readily (dark green symbols) predominantly cluster within the “T + K” quadrant, indicating that such forms benefit from both thermodynamic stability and favourable kinetic factors. In contrast, crystal forms described as more difficult to crystallise systematically exhibit higher lattice energies and/or higher particle rugosities, reducing their overall advantage. Importantly, however, these forms still predominantly occupy the “K” or “T + K” quadrants, indicating that they remain experimentally accessible under appropriate conditions.

This behaviour is particularly evident within the carbamazepine family of compounds (Fig. 8b–d). The more difficult-to-crystallise polymorphs (oxcarbazepine form III, dihydrocarbamazepine formIV, and carbamazepine forms V, IV, and II) are concentrated primarily in the “K” quadrant, characterised by relatively lower rugosities but higher lattice energies. Among these, carbamazepine form V lies close to the boundary of the “T + K” quadrant, consistent with its marginal experimental accessibility.

Notably, across all six systems, no experimentally observed crystal structures occupy either the “T” or “X” quadrants outside the vertical pink zone of expected rugosity with MAD. Several tolfenamic acid polymorphs approach the upper limit of the experimental rugosity band yet still remain within kinetically accessible regions. The overwhelming majority of experimental structures fall within the experimentally defined rugosity band, with the few exceptions predominantly located in the “T + K” quadrant. This observation supports the value of combining lattice-energy ranking with rugosity-based landscape partitioning as an indicator of experimental accessibility.

The tolfenamic acid landscape provides a particularly illustrative example: the thermodynamically most stable predicted structure exhibits very high particle rugosity and lies squarely within the “T” quadrant. This form has not been observed experimentally, which may be attributed to kinetic limitations associated with nucleation and growth stemming from its rough crystal surfaces.

When comparing polymorphic and monomorphic systems, no systematic differences emerge in the overall topology of the CSP landscapes. In particular, particle rugosity alone does not appear to provide a reliable apriori indicator of whether a system will exhibit polymorphism. More sophisticated analyses—such as investigations of structural relationships, transformation pathways, and finite-temperature effects—may be required to address this question^56,57^.

Overall, particle rugosity emerges as a valuable ranking descriptor for assessing the relative experimental accessibility of predicted crystal structures within CSP landscapes. In particular, the upper bound of the experimental rugosity band appears to provide an indicator of reduced experimental accessibility via crystallisation. Incorporating rugosity alongside lattice energy within CSP workflows enables the prioritisation of structures that are both thermodynamically viable and kinetically accessible, improving the efficiency and interpretability of polymorph prediction, with potential implications for the early-stage risk identification for solid forms in pharmaceutical development. At the same time, the imperfect correlation between low rugosity and ease of crystallisation underscores the necessity of a multi-descriptor approach, that integrates rugosity with hydrogen-bonding metrics, thermodynamic factors, conformational energies and solvent effects to better bridge the gap between computed landscapes and experimental outcomes.

## Conclusions

This work introduces a computational surface analysis tool for molecular crystals that enables quantitative assessment of particle rugosity as a descriptor of crystallisation behaviour. Across polymorphic families, CSP-generated landscapes, and large subsets of the CSD, particle rugosity emerges as a useful complementary descriptor and ranking aid for experimental accessibility. Structures with lower rugosity values are generally associated with easier crystallisation, either through enhanced nucleation or uninhibited growth kinetics, while high-rugosity surfaces tend to correlate with growth inhibition and increased kinetic barriers.

While particle rugosity is a promising descriptor, it should not be considered a standalone predictor of crystallisation behaviour. Rather, it is most effective when used as part of a multi-descriptor framework, complementing established approaches such as lattice energy calculations, conformational energies, hydrogen-bonding analyses, and solvent–surface interaction studies. Statistical analysis of rugosity trends across CSD structures further reveals size-dependent accessibility bands, which enable CSP landscapes to be partitioned into regions associated with differing likelihoods of experimental realisation. This offers a practical strategy for guiding polymorph screening and reducing the search space for experimental realisation.

Particle rugosity therefore provides a physically intuitive link between molecular-scale surface topology and macroscopic crystallisation behaviour, and can serve as a helpful classifier within crystal structure prediction workflows. Future work will focus on integrating rugosity with complementary molecular and thermodynamic descriptors  (e.g. conformational energies) to^39,40^ develop more robust and transferable models of crystallisation. To support uptake by the community, the Python script used for particle rugosity calculations is made openly available via a GitHub repository.

## Supplementary information

Supplementary Material Rugosity

## Data Availability

The authors declare that the data supporting the findings of this study are available within the paper and its supplementary information file. The full dataset and code developed have been deposited in the Durham University Data Repository under 10.15128/r1w0892b031.

## Code availability

The surface properties code is available on Github at the following link: https://github.com/DuMOCC-Group/surface_rugosity.git. The code is also available within the full dataset deposited in the Durham University Data Repository under 10.15128/r1w0892b031.
