# Supplementary material for: Assessing crystallisation behaviour in molecular crystals through particle rugosities
Source: Commun Chem. 2026 Jun 29;9:237. doi: 10.1038/s42004-026-02104-5 (PMC13346652; doi:10.1038/s42004-026-02104-5)
Supplement: Supplementary file 1 — Supplementary Material Rugosity [file 42004_2026_2104_MOESM1_ESM.pdf]

SUPPORTING INFORMATION:

Assessing Crystallisation Behaviour in Molecular Crystals through Particle Rugosities

Marta Brocca[a], Dominic Evans[a], Helen Blade[b], Sten O. Nilsson Lill[c], Aurora J. Cruz-Cabeza[a*]

a. Department of Chemistry, University of Durham, South Road, Durham, DH1 3LE, UK
b. Global Product Development, Pharmaceutical Technology & Development, Operations, AstraZeneca Macclesfield, SK10 2NA, Cheshire, UK
c. Predictive Science, Digital & Automation, Pharmaceutical Sciences, R&D, AstraZeneca Gothenburg, Mölndal, Sweden

*Corresponding author: aurora.j.cruz-cabeza@durham.ac.uk

The supplementary material contains detailed information about the CSP landscapes presented in the main article (section S1), details of the optimisation of the offset resolution *n* (section S2), the distribution of modal rugosity values across CSD systems having at least one HB donor per molecule (section S3), the correlation between particle rugosity and molecular size single component organic structures (section S4), and a complete figure of the CSP landscapes analysis for the all the three offsets (section S5).

S1. Datasets – CSP landscapes

The details of the CSP landscapes analysed with the new Python scripts are presented here. The .cif files of the predicted structures were kindly provided to us by Prof. S. L. Price and her research group. CSP results are presented in the main paper in section 4.6.

Table S 1 CCDC refcodes and literature references for the CSP landscapes presented in this work

Polymorphic systems			Monomorphic systems		
**REFCODE**	**Name**	**Reference**	**REFCODE**	**Name**	**Reference**
CANDUR	Oxcarbazepine	1	QQQBTY	Fenamic Acid	2
CBMZPN	Carbamazepine	3	SCCHRN	Saccharin	4
KAXXAI	Tolfenamic Acid	5			
VACTAU	Dihydrocarbamazepine	3			

S2. Variable Selection for Particle Rugosity Calculations

Figure S 1 shows the effect of varying the offset resolution n on the density of unsatisfied hydrogen bond (HB) donors at the apolar and polar offset. Similarly to the results obtained for the [s] offset (Figure 3 in the main paper), convergence is achieved for all systems at $n \geq 4$, with an exception for Ritonavir (refcode YIGPIO03, red line) for the [p] offset. This behaviour arises from the large size and conformational flexibility of the molecule, for which small changes in the offset can produce distinct surface terminations and functional group exposures. Increasing n enhances the sampling of these configurations and, in such cases, can lead to the identification of surface terminations with more extreme hydrogen-bonding characteristics, resulting in increased variability. This effect is structure-dependent and more pronounced for larger, flexible molecules, which also exhibit greater variation in particle rugosity (see Figure 7 in the main manuscript). Overall, $n = 4$ provides a practical compromise between sampling resolution, robustness, and computational efficiency.

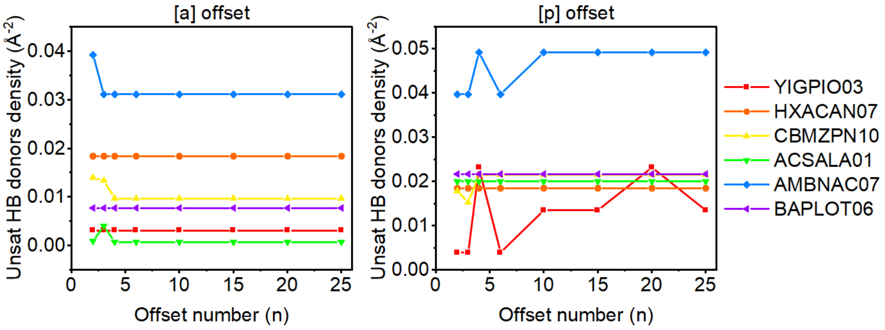

Figure S 1 Influence of the offset resolution n on the density of unsatisfied HB donors for the [a] and [p] offset data. Results are reported for six representative systems, identified by their CSD refcodes in the legend at the right of the figure. All calculations were performed using the BFDH morphology model.

S3. Rugosity trends in molecular crystals – only HB donors

Here we present the distribution of modal rugosity values across two large CSD datasets (more info in the main paper, section 4.5). Differently from the analysis shown in the main text (Figure 6), here only the systems with at least one HB donor per molecule are considered.

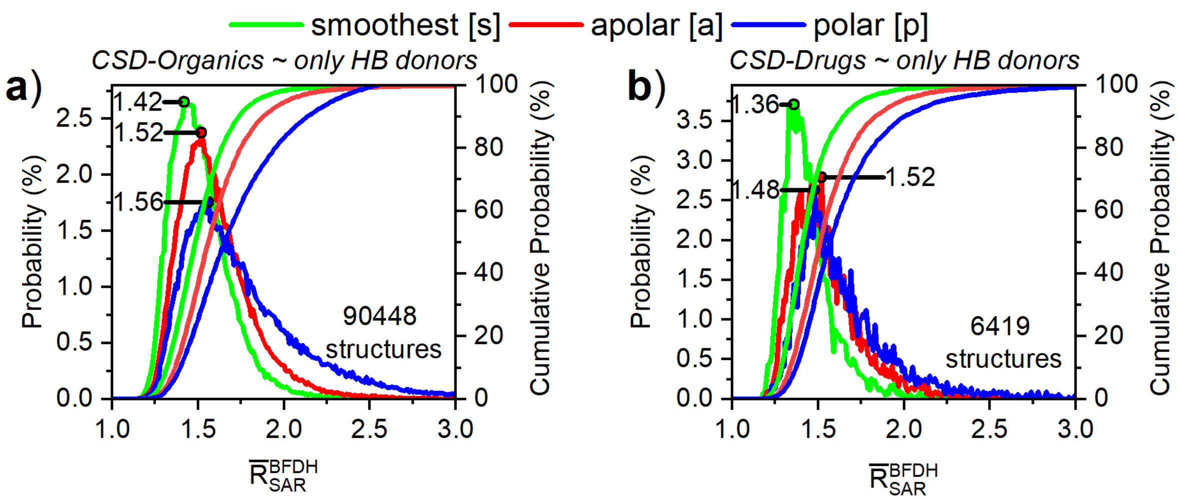

Figure S 2 Distribution of $\overline{R}_{SAR}^{BFDH}$ in the CSD on different datasets – a) "best R-factor" list and b) the "CSD-drug-subset". Structures with no HB donors were removed from these datasets to investigate the changes in the rugosity distributions

S4. Rugosity trends in molecular crystals – single component structures

Here we provide the analogue analysis that is presented in the main text in section 4.5 (Figure 7), restricted to single-component only structures that belong to dataset 4 (CSD-Organics). The total number of structures analysed here is 124,090. The single-component-only trend is comparable to the wider one presented in Figure 7, with parameters in the fitting equations only slightly varying.

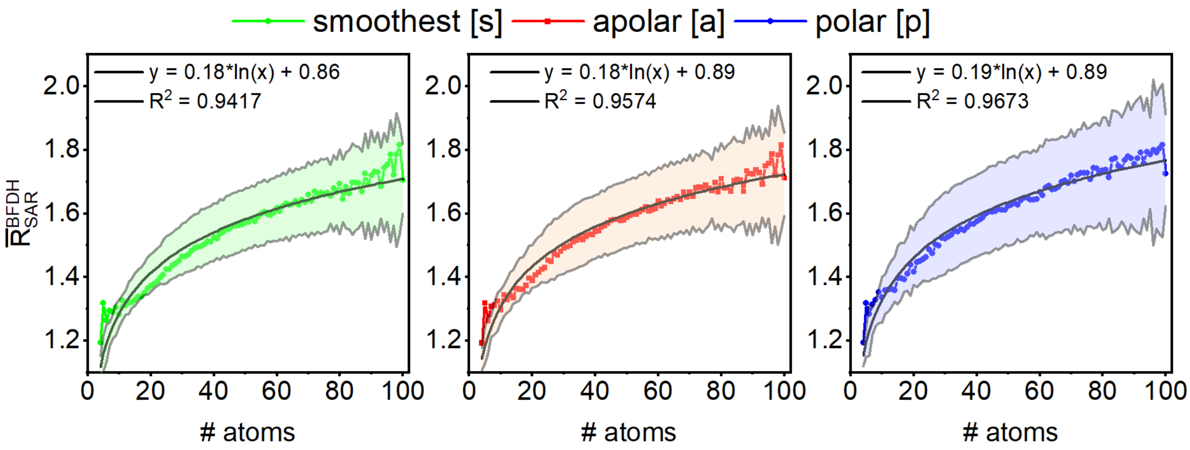

Figure S 3 Correlation between particle rugosity ($\bar{R}_{SAR}^{BFDH}$) and molecular size (number of atoms per molecule) for *single component* structures in Dataset 4.

S5. Crystal Structure Prediction

Here all the 6 analysed CSP landscapes are presented (Figure S3). The landscapes were divided into quadrants as explained in section 4.6 of the main paper. For each system, the rugosities obtained at the three possible offsets (smoothest, apolar and polar) are reported.

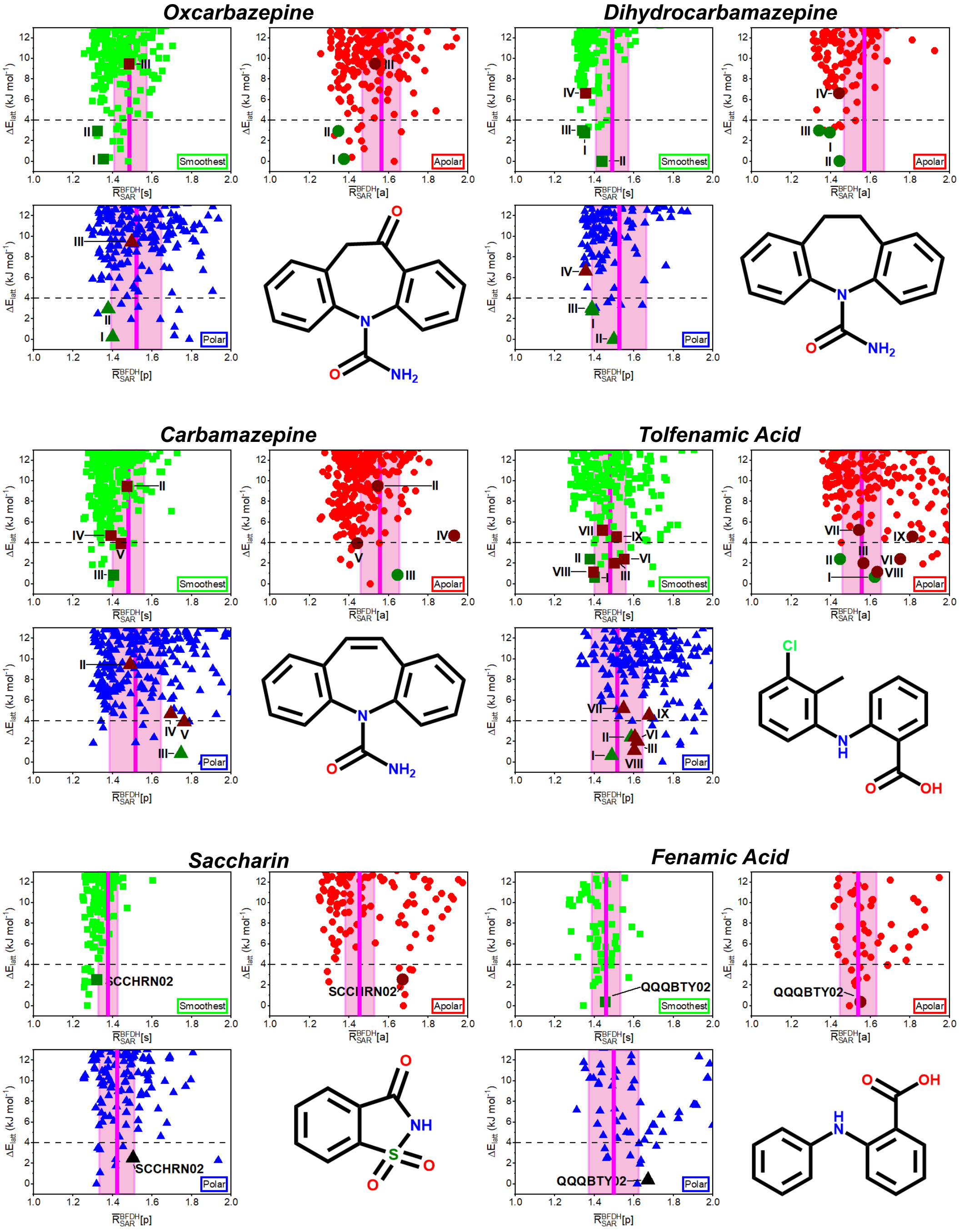

Figure S 4 CSP landscapes (relative lattice energy vs particle rugosity) for six systems (the first four polymorphic and the last two monomorphic). For the polymorphic systems, experimentally accessible structures are labelled in green, whereas structures that are difficult to crystallise are shown in red. The quadrant division follows the same scheme as described in the main text. For each structure, the landscape includes calculations performed at each rugosity offset ([s], [p], and [a]).

References

1 Polyzois, H. *et al.* Crystal Structure and Twisted Aggregates of Oxcarbazepine Form III. *Cryst. Growth Des.* **22**, 4146-4156 (2022).

2 Uzoh, O. G., Cruz-Cabeza, A. J. & Price, S. L. Is the Fenamate Group a Polymorphophore? Contrasting the Crystal Energy Landscapes of Fenamic and Tolfenamic Acids. *Cryst. Growth Des.* **12**, 4230-4239 (2012).

3 *Control and Prediction of the Organic Solid State*, <https://www.chem.ucl.ac.uk/cposs/index.htm> (2019).

4 Corpinot, M. K. *et al.* Are Oxygen and Sulfur Atoms Structurally Equivalent in Organic Crystals? *Cryst. Growth Des.* **17**, 827-833 (2017).

5 Case, D. H. *et al.* Successful Computationally Directed Templating of Metastable Pharmaceutical Polymorphs. *Cryst. Growth Des.* **18**, 5322-5331 (2018).
